# CTEN Induces Tumour Cell Invasion and Survival and Is Prognostic in Radiotherapy-Treated Head and Neck Cancer

**DOI:** 10.3390/cancers12102963

**Published:** 2020-10-13

**Authors:** Jason C. Fleming, Jeongmin Woo, Karwan Moutasim, Christopher J. Hanley, Steven J. Frampton, Oliver Wood, Matthew Ward, Christopher H. Woelk, Christian H. Ottensmeier, Sassan Hafizi, Dae Kim, Gareth J. Thomas

**Affiliations:** 1 Cancer Sciences, Faculty of Medicine, University of Southampton, Southampton SO17 1BJ, UK; K.A.Moutasim@soton.ac.uk (K.M.); C.J.Hanley@soton.ac.uk (C.J.H.); Steven.frampton@poole.nhs.uk (S.J.F.); O.Wood@soton.ac.uk (O.W.); matthew.ward@porthosp.nhs.uk (M.W.); c.ottensmeier@liverpool.ac.uk (C.H.O.); 2 Liverpool Head & Neck Centre, University of Liverpool, Liverpool L3 9GA, UK; 3 Liverpool University Hospitals NHS Foundation Trust, Liverpool L9 7AL, UK; 4 Clinical and Experimental Sciences, Faculty of Medicine, University of Southampton, Southampton SO17 1BJ, UK; jeongmin.woo@rdm.ox.ac.uk (J.W.); christopher.woelk@merck.com (C.H.W.); 5 Exploratory Science Center, Merck & Co., Inc., Cambridge, MA 02141, USA; 6 Clatterbridge Cancer Centre NHS Foundation Trust, Liverpool CH63 4JY, UK; 7 School of Pharmacy & Biomedical Sciences, University of Portsmouth, Portsmouth PO1 2DT, UK; sassan.hafizi@port.ac.uk; 8 St. George’s University Hospitals NHS Foundation Trust, Tooting, London SW17 0QT, UK; Dae.Kim@stgeorges.nhs.uk

**Keywords:** head and neck cancer, tensin, CTEN, invasion, apoptosis, radiotherapy, biomarker, human papillomavirus

## Abstract

**Simple Summary:**

C-terminal tensin-like, or CTEN, is a cytoskeletal protein that is expressed highly in head and neck cancer (HNSCC). We studied CTEN function using gene knockdown and found that CTEN contributes to HNSCC progression in several ways, promoting tumour cell invasion and also cell survival. Notably, CTEN expression protects tumour cells from radiation-induced apoptosis and consistent with this, we found that CTEN expression predicts for survival in patients treated with radiotherapy (but not surgery), suggesting that CTEN may have utility as a predictive marker of radiotherapy resistance.

**Abstract:**

Head and neck squamous cell carcinoma (HNSCC) is a heterogenous disease treated with surgery and/or (chemo) radiotherapy, but up to 50% of patients with late-stage disease develop locoregional recurrence. Determining the mechanisms underpinning treatment resistance could identify new therapeutic targets and aid treatment selection. C-terminal tensin-like (CTEN) is a member of the tensin family, upregulated in several cancers, although its expression and function in HNSCC are unknown. We found that CTEN is commonly upregulated in HNSCC, particularly HPV^−ve^ tumours. In vitro CTEN was upregulated in HPV^−ve^ (*n* = 5) and HPV^+ve^ (*n* = 2) HNSCC cell lines. Stable shRNA knockdown of CTEN in vivo significantly reduced tumour growth (SCC-25), and functional analyses in vitro showed that CTEN promoted tumour cell invasion, colony formation and growth in 3D-culture (SCC-25, Detroit 562). RNA sequencing of SCC-25 cells following CTEN siRNA knockdown identified 349 differentially expressed genes (logFC > 1, *p* < 0.05). Gene ontology analysis highlighted terms relating to cell locomotion and apoptosis, consistent with in vitro findings. A membrane-based antibody array confirmed that CTEN regulated multiple apoptosis-associated proteins, including HSP60 and cleaved caspase-3. Notably, in a mixed cohort of HPV^+ve^ and HPV^−ve^ HNSCC patients (*n* = 259), we found a significant, independent negative association of CTEN with prognosis, limited to those patients treated with (chemo)radiotherapy, not surgery, irrespective of human papillomavirus (HPV) status. These data show that CTEN is commonly upregulated in HNSCC and exerts several functional effects. Its potential role in modulating apoptotic response to therapy suggests utility as a predictive biomarker or radio-sensitising target.

## 1. Introduction

With over 500,000 new diagnoses each year and a mortality over 50% [1], the disease burden of head and neck cancer squamous cell carcinoma (HNSCC) is significant. Survival rates have remained relatively unchanged over the past few decades, although the main treatment modalities of surgery and radiotherapy, with or without concomitant chemotherapy, have resulted in an overall 60–90% chance of cure with early stage disease [2]. However, prognosis continues to be highly dependent on locoregional tumour burden, and late presentation of disease accounts for over 60% of new diagnoses [3]. Whilst there is promise with new large genomic studies to identify potential novel disease biomarkers and therapeutic targets [4], there has been a dearth of clinical studies translating promising pre-clinical work to investigate novel prognostic factors in this disease. 

Human papillomavirus (HPV) status is the only molecular stratifier used routinely in current clinical practice [5] (mostly identified by using p16 expression as a surrogate marker) and is the single most significant non-anatomical prognostic marker [6]. Despite a higher incidence of positive lymph node involvement at presentation, which tended to skew staging information in the American Joint Committee on Cancer (AJCC) 7th Edition Manual towards advanced disease [7], patients with HPV^+ve^ tumours have repeatedly been shown to have improved disease-free and overall survival compared to those with HPV^−ve^ disease [8,9,10], although notably, a significant subset have more aggressive disease with poor clinical outcome [11]. This was addressed in the recent 8th Edition of the AJCC TNM Staging Manual by ratifying and downstaging multiple node groups within a separate HPV^+ve^ category [12]. The difference in prognosis is at least partly due to the increased radio/chemosensitivity of HPV^+ve^ tumours [13], but there also other major biological differences between these tumour subtypes, with HPV^+ve^ tumours commonly retaining wild-type p53 and Rb gene modulation and showing an absence of field cancerisation and an increased host adaptive immune responses; all subjects of recent research [14,15,16]. Following the recognition of the long-term treatment-related morbidity effects of radiotherapy, especially when combined with concurrent chemotherapy for advanced disease [17], there has been a drive for minimally invasive surgical techniques via a trans-oral route, with over 80% patients in the US, for example, now undergoing primary surgery for early stage (T1-2), lateralised disease [18]. A number of clinical trials are also investigating de-escalation protocols for HPV^+ve^ tumours. However, to avoid under-treatment and potential harm to the minority of patients harbouring a more aggressive disease phenotype, research on markers and mechanisms of radiosensitivity are urgently required. 

Tensins are focal adhesion adaptor proteins that have recently been implicated in the progression of a variety of cancer types, with C-terminal tensin-like (CTEN) receiving the most attention. As well as exhibiting a relatively restricted pattern of expression in normal human tissues [19], CTEN appears to be the sole Tensin family member that mediates an oncogenic effect [20,21,22,23,24,25], with studies showing that it regulates integrin-mediated cell motility [22], is involved in a variety of signalling pathways [26,27] and also modulates Rho GTPase-activating proteins [28,29], suggesting that CTEN may broadly promote tumour cell invasion and metastasis [30]. Tumour invasion and locoregional recurrence have a major impact on head and neck cancer patient survival, with both invasion depth [31] and pattern [32] being prognostic features. The function and clinical importance of CTEN in HNSCC has not previously been investigated. In this study, we demonstrate that CTEN is highly expressed in HNSCC, specifically in HPV^−ve^ disease. CTEN expression supports tumour growth in both in vitro and in vivo models through effects on invasion and cell survival, potentially representing a marker of radiosensitivity and a therapeutic target.

## 2. Results

### 2.1. CTEN Expression in HNSCC

**Figure 1:**
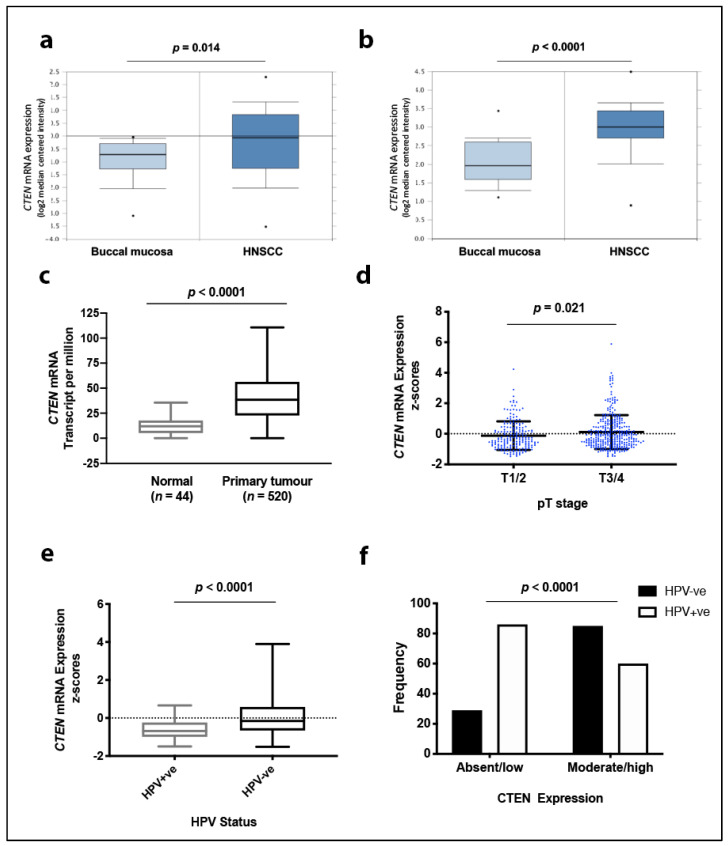
C-terminal tensin-like (CTEN) expression in head and neck squamous cell carcinoma (HNSCC) databases. mRNA expression levels of CTEN were significantly higher in head and neck cancer than in normal tissue across databases, as illustrated in boxplots including the (**a**) Ginos Head-Neck (*n* = 54), (**b**) Peng Head-Neck (*n* = 79) and (**c**) The Cancer Genome Atlas (*n* = 564) datasets. Subgroup analysis of tumour samples in The Cancer Genome Atlas (TCGA) (*n* = 520) demonstrates higher CTEN expression in advanced T-stage compared with early stage disease (**d**) and in human papillomavirus (HPV)^−ve^ (*n* = 243) compared with HPV^+ve^ disease (*n* = 36, [**e**]). Box plot length indicates min. to max. range and central line for the mean value and *p*-values (Student’s *t*-test) indicated above the graphs. (**f**) Immunohistochemical analysis of our oropharyngeal squamous cell carcinoma database (*n* = 259; HPV^−ve^ *n* = 113; HPV^+ve^ *n* = 146) corroborates a greater CTEN protein expression in HPV^−ve^ tumour specimens (Chi squared, χ^2^ = 29.06).

To investigate expression of CTEN in HNSSC, we first interrogated several publicly available HNSCC datasets. Analysis of the Oncomine database [33] and both the Peng Head-Neck [34] and Ginos Head-Neck [35] datasets revealed that mRNA expression of CTEN was significantly higher in HNSCC tissue samples compared with normal tissue (fold change 1.691 (*p* = 0.014) and 1.894 (*p* < 0.0001), respectively; Figure 1a,b). Analysis of the Pyeon multi-cancer database [36] showed that, of all the head and neck subsites, tonsillar carcinoma (oropharyngeal cancer) showed the greatest differential change compared to non-tumour tissue (fold change 1.581). HNSCC samples in The Cancer Genome Atlas (TCGA) directory (*n* = 520; [4]) also showed increased CTEN mRNA expression in tumour tissue compared to normal controls (*p* < 0.0001, Figure 1c), with expression significantly higher in advanced primary disease (pT3/4) compared with early stage disease (pT1/2, *p* = 0.021; Figure 1d), and in HPV-negative disease compared with virally-derived tumours (*p* < 0.0001; Figure 1e). We were able to confirm this finding at the protein level in a large oropharyngeal squamous cell carcinoma (OPSCC) tumour cohort (*n* = 259; HPV^−ve^ *n* = 113; HPV^+ve^ *n* = 146) [16], with immunohistochemistry similarly demonstrating moderate/strong CTEN expression in 74.6% of HPV^−ve^ tumours compared with 41.1% of HPV^+ve^ tumours (*p* < 0.0001; Figure 1f). Notably, further analysis of TCGA data revealed that HNSCC shows higher CTEN expression than any other tumour type (Figure S1). 

### 2.2. CTEN Depletion Reduces Tumour Growth In Vivo

**Figure 2:**
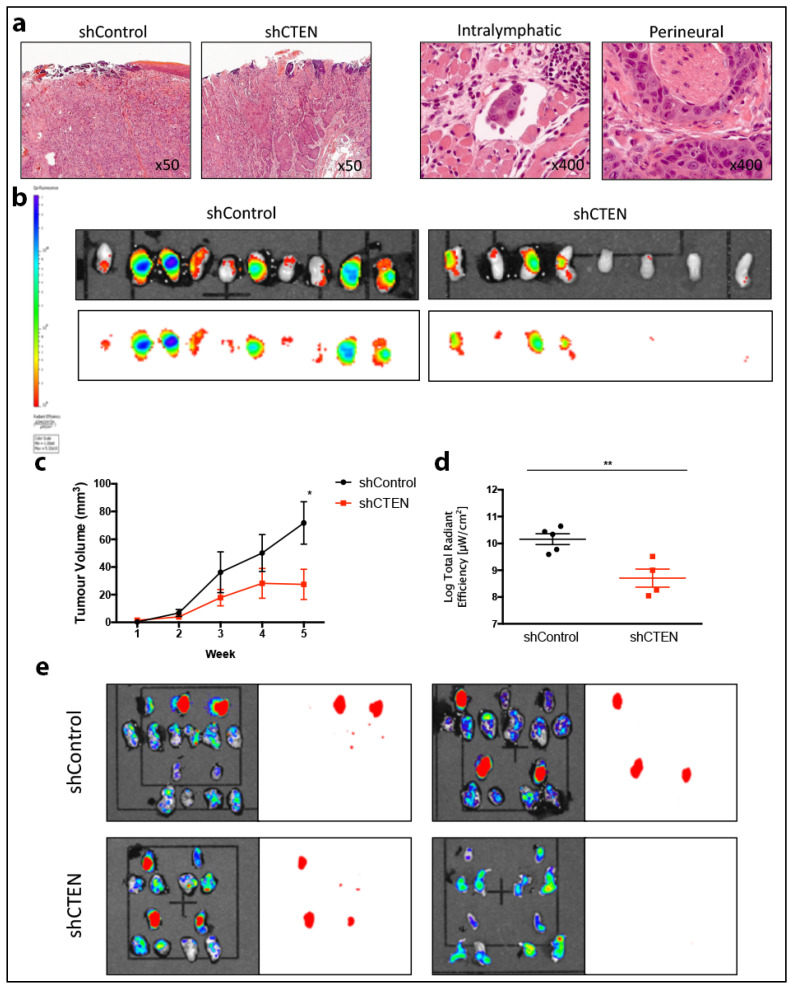
CTEN knockdown reduces tumour growth in an orthotopic mouse model of oral cancer. Xenograft tumours were made by orthotopically injecting shCtrl- or shCTEN-treated SCC25 cells, allowed to grow for 6 weeks with microcalliper measurement weekly and then dissected and pathologically examined. The orthotopic tumours mimicked the pathological appearance of human HNSCC accurately, including other features of aggressive tumour invasion, such as lymphatic and perineural invasion in both the control and CTEN-knockdown tumours (**a**). (**b**) Post-mortem tongue specimens were dissected and imaged on a small animal imager utilising a GFP tag included with the shRNA vectors. These are displayed as a GFP/photo overlay (upper panel) or GFP subtraction image only (bottom panel). Microcalliper tumour volumes (**c**) and automated measured radiant efficiencies (**d**) of tongue tumour samples corroborated a significant tumour growth reduction in CTEN knockdown tumours (Student’s *t*-test, * *p* < 0.05, ** *p* < 0.01). (**e**) Tongue specimens (top specimen in each double row) and bilateral cervical lymph node chains (bottom specimens (two per animal) in each row) were surgically dissected and imaged for GFP expression. The subsequent image overlays then underwent digital image subtraction to identify tumour deposits in the cervical lymph node biopsies. shControl cells resulted in 5/10 primary tumours compared with 6/10 clinically or image evident tumours in shCTEN cells. However, there were six regional lymph node deposits in shControl versus only three in shCTEN models. Two mouse specimens were not included in the final imaging analysis due to their demise in the week prior to experiment completion. Their results up to the point of death have been included in the growth analysis.

We next investigated expression of CTEN in a panel of human HNSCC cell lines (Figure S2). All HNSCC cell lines (HPV^−ve^ = 5; HPV^+ve^ *n* = 2) expressed CTEN at similar or higher levels to MCF7, a breast cancer cell line used as a positive control tumour type for CTEN expression [22]. To study the effect of CTEN expression on tumour growth in vivo, we generated a stable CTEN shRNA knockdown of SCC-25 cells, also expressing a GFP tag. Following selection of a mixed cell population with >90% knockdown, we tested CTEN function in vivo using an orthotopic oral HNSCC mouse model. Tumours showed typical features of HNSCC, infiltrating into the tongue musculature and showing patchy keratin formation as well as intralymphatic and perineural infiltration (Figure 2a, right panel); no gross differences in tumour architecture were noted between the control and test populations. CTEN knockdown resulted in significantly reduced tumour growth (*p* < 0.05 at 5 weeks; Figure 2b–d), without any subjective difference in tumour morphology, and a reduction in micrometastases (six (shCtrl) vs. three (shCTEN); Figure 2b,e).

### 2.3. CTEN Supports Head and Neck Cancer Progression through Effects on Both Invasion and Survival

**Figure 3:**
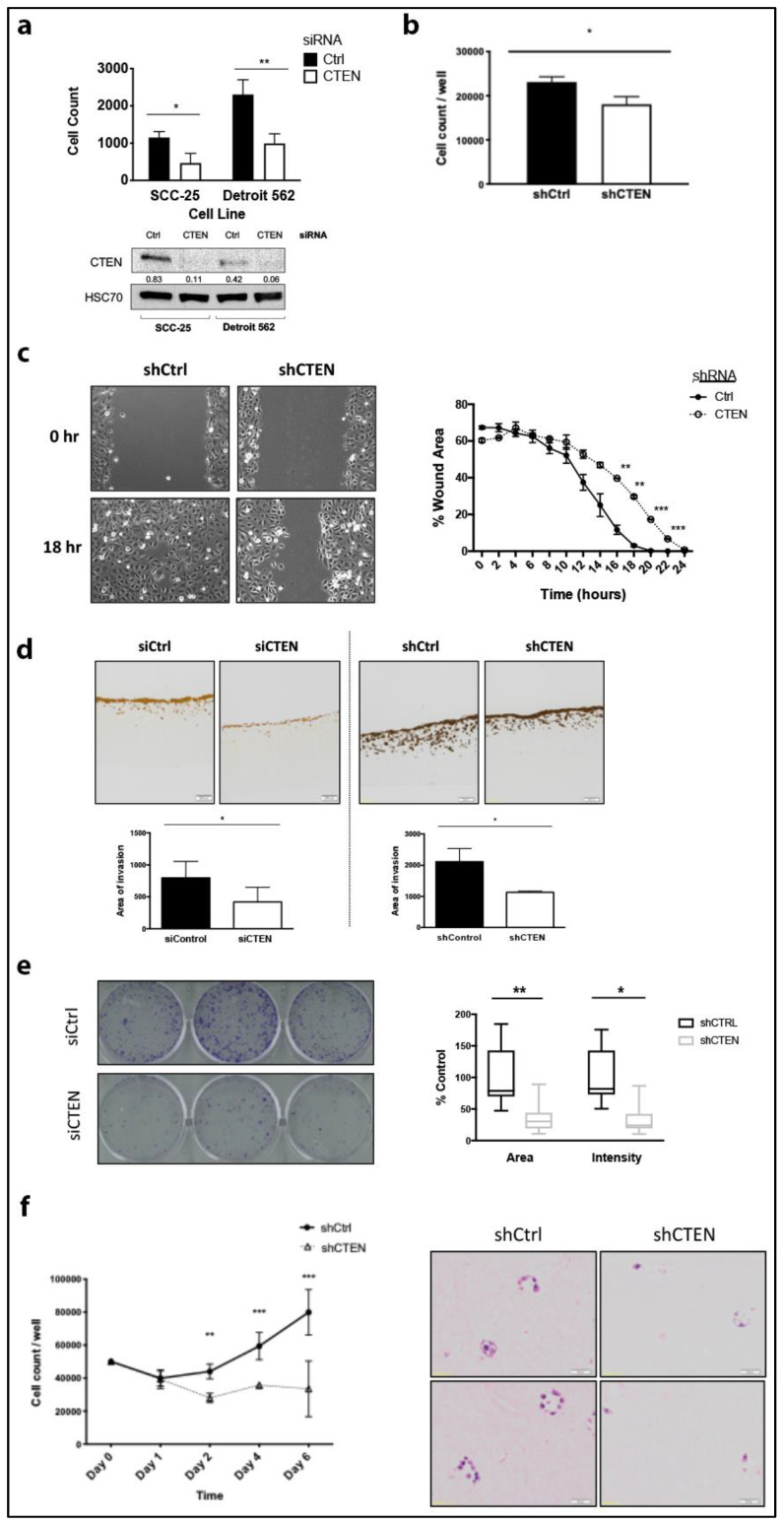
CTEN supports head and neck cancer progression through effects on both invasion and survival. (**a**) Transient CTEN knockdown significantly reduced invasion of SCC-25 (* *p* < 0.05; Student’s *t*-test) and Detroit 562 (** *p* < 0.01) cell lines across a Matrigel^TM^ layer in Transwell inserts towards serum-containing media in the bottom chamber over 72 h. Western blots confirming knockdown are presented underneath corresponding cell lines (lower panel) and HSC70 was used as a loading control. (**b**) Similar effects of reducing cell motility were observed in stable CTEN-knockdown SCC-25 cells (* *p* < 0.05) in a migration assay performed over 48 h. (**c**) Time-lapse microscopy revealed an inhibition of SCC-25 motility in CTEN-knockdown cells on a 24-h scratch wound-healing assay. Images were analysed for wound coverage area on ImageJ [37] using TScratch software [38] and the Chemotaxis plugin for ImageJ (Ibidi GmbH), revealing a significant delay in wound closure in CTEN-knockdown cells (Student’s *t*-test, two-tailed; ** *p* < 0.01; *** *p* < 0.001). (**d**) Organotypic cultures using either transient (left panel) or stable CTEN-knockdown (right panel) SCC-25 cells with HFFF2 cells show significant reduction in the cell invasion area in both cell lines compared to controls (* *p* < 0.05; paired *t*-test). Representative pancytokeratin stained sections (4 μm) are demonstrated with 40x magnification. (**e**) Clonogenic assays demonstrated the effect of CTEN inhibition on SCC-25 cells. Results are presented relative to % control well. CTEN knockdown produced a significant reduction in both colony area and intensity (* *p* < 0.05, ** *p* < 0.01, respectively). Representative images are shown, and analysis was performed utilising ColonyArea plugin [39] on ImageJ. (**f**) 3D collagen gel proliferation assays performed on control shRNA-transduced or stable CTEN-knockdown cells suspended in a collagen gel demonstrated a significant increase in proliferation in the control cells compared to knockdown cells (** *p* < 0.01, *** *p* < 0.001; Student’s *t*-test). A gel from each condition at day 6 was fixed and processed for staining with H&E, demonstrating no 3D cell-cell contracts or cell groupings in the knockdown cultures.

In vivo results showed that CTEN promotes local tumour growth and metastases. To investigate further the functional role of CTEN, we performed a series of in vitro experiments, transiently and stably silencing CTEN expression then examining cell invasion, migration and colony-forming ability. Silencing CTEN significantly reduced invasion of SCC-25 and Detroit 562 HNSCC cell lines (Figure 3a,b and Figure S3) but had no significant effect on cell proliferation over these short, monolayer 72-h assays (Figure S4). Cell migration in scratch assays was also reduced following CTEN knockdown (Figure 3c). 

To further investigate the effect of CTEN on cell invasion, we used a more physiologically relevant, 3D organotypic invasion model incorporating HFFF2 fibroblasts, which have previously been used to study HNSCC invasion [40]. CTEN knockdown significantly inhibited invasion (*p* < 0.05; Figure 3d), reducing both the depth of invasion and the number of invading tumour islands.

We next tested the effect of CTEN knockdown on clonogenic growth of tumour cells by performing colony forming assays (Figure 3e) and 3D cell proliferation assays (Figure 3f) over 10 and 6 days, respectively. Notably, CTEN silencing significantly reduced both the number and size of cell colonies formed and also inhibited 3D culture colony growth, suggesting a CTEN-dependent pro-proliferative/survival effect. 

### 2.4. Loss of CTEN Expression Promotes Apoptosis

**Figure 4:**
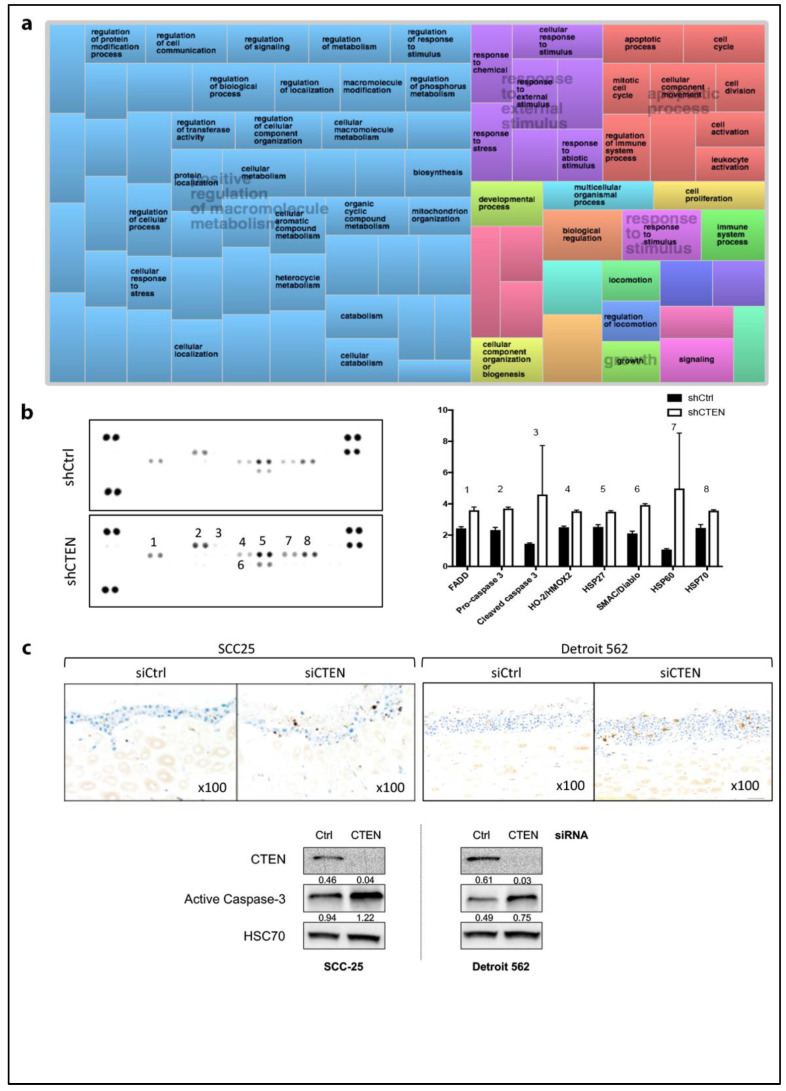
CTEN expression promotes apoptotic pathways. (**a**) TreeMap visualisation obtained from REVIGO [44] analysis of the summary of gene ontology terms for all differentially expressed genes (*p* < 0.01) between control and CTEN siRNA-treated samples, with ‘Apoptotic Process’ domain figuring prominently (top right). Related ontology terms with semantic similarity are grouped in the same colours and the dimensions of the coloured areas are proportional to the direction of the impact. Not all terms are shown due to space constraints. (**b**) The expression of 39 proteins involving in apoptosis regulation were determined in cell lysates from stable CTEN-knockdown or control cells, using the Proteome Profiler™ Array—Human Apoptosis Array Kit (R&D Systems), according to the manufacturer’s instructions. Signals obtained were quantified using ImageJ software [37] and presented as expressions relative to endogenous controls (representative array presented of two biological repeats). (**c**) Organotypic culture sections from transient CTEN-knockdown SCC25 and Detroit 562 assays were stained for activated caspase-3. Representative micrographs for SCC25 and Detroit 562 are demonstrated (×100 magnification, upper panels). Increased caspase-3 expression is evident across cell lines in CTEN siRNA-treated cells. Western blot results corroborate caspase-3 activation in CTEN-knockdown cells (lower panels). HSC70 was utilised as loading control.

In vitro results suggested that CTEN may regulate several cell functions. In order to investigate this further, we performed high-throughput RNA sequencing analysis on control vs. CTEN siRNA-treated SCC-25 cells. A total of 349 differentially expressed genes (DEGs) were identified (234 up-regulated and 115 down-regulated genes (logFC > 1, *p* < 0.05)), distilled into a gene ontology hierarchy visualisation matrix. In keeping with our in vitro results, we observed prominent terms relating to ‘cell locomotion’ and ‘signalling’ and a larger cluster under ‘response to external stimulus’. Additionally, in support of a CTEN-dependent effect on cell proliferation/survival, a large cluster under ‘apoptotic processes’ featured prominently (Figure 4a, upper right quadrant). To investigate apoptosis further, we performed a membrane-based antibody array following CTEN silencing (Figure 4b). Analysis demonstrated that loss of CTEN was associated with the upregulation of multiple apoptosis-related proteins, particularly the molecular chaperone HSP60 and cleaved (activated) caspase-3. To corroborate these findings, we performed immunohistochemistry for activated caspase-3 on SCC-25 and Detroit 562 organotypic culture sections (Figure 4c, Figure S5). We observed markedly increased expression of activated caspase-3 in the CTEN-knockdown cancer cell cultures compared to control siRNA-treated paired samples (median positive staining % nuclei per high power field: 5.19 (siCtrl, IQR 4.94–6.16) vs. 0.85 (siCTEN, IQR 8.46–10.14), *p* = 0.0027, respectively) with Western blot confirmation. Ow et al [41] found, in two independent datasets, that BCL2L1 and MCL1 are the most significantly elevated anti-apoptotic markers in HNSCC. We therefore analysed the TCGA HNSCC database for correlation between CTEN and pro-apoptotic CASP3 and anti-apoptotic BCL2L1 and MCL1 [42,43] (Figure S6). This corroborated our results, demonstrating a significant negative correlation between CTEN and CASP3 (Spearman’s rho = −0.29, *p* < 0.0001) and a corresponding positive correlation between BCL2L1 (0.15, *p* = 0.0013) and MCL1 (0.37, *p* < 0.0001).

### 2.5. CTEN Is a Prognostic and Radiosensitivity Marker in HNSCC

**Figure 5:**
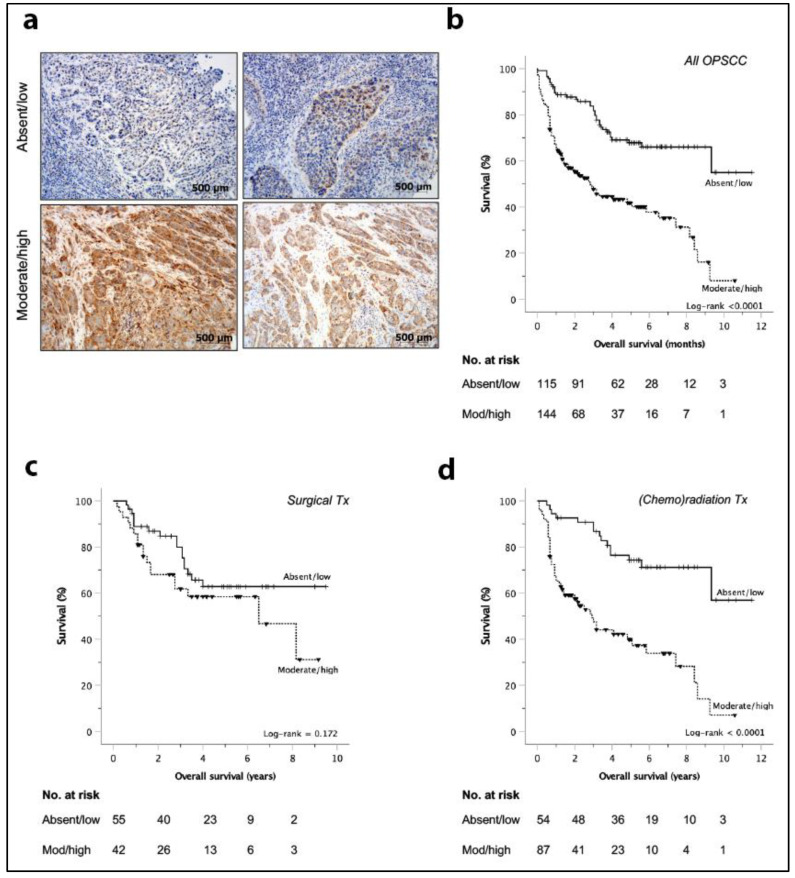
Survival analysis for oropharyngeal squamous cell carcinoma (OPSCC) dataset. (**a**) Representative immunohistochemistry slides showing examples of CTEN expression levels in HPV^−ve^ and HPV^+ve^ HNSCC. Scale bar = 500 microns. (**b**–**d**) Kaplan–Meier overall survival curves for all oropharyngeal SCC (*n* = 259) with CTEN expression scored on immunohistochemistry as low (absent/weak) or high (moderate/high): (**b**) demonstrating a significant correlation between CTEN expression and overall survival in OPSCC (log-rank < 0.0001). Analysis by primary treatment modality demonstrated that the surgery group (**c**) failed to show an association with CTEN expression (log-rank = 0.172) whereas the cohort undergoing organ sparing treatment modalities (**d**) (chemo) radiation) again showed a significant correlation between CTEN expression and overall survival (log-rank < 0.0001).

With the demonstrated pleiotropic effects of CTEN expression that enhance cancer cell invasion and survival, we sought to explore the potential clinical relevance of this protein in a retrospective cohort. Immunohistochemistry analysis of a large HNSCC cohort (*n* = 259 with full survival data; Table 1) demonstrated that CTEN expression was negatively associated with overall survival (OS) (*p* < 0.0001, Figure 5a,b), with a 5-year OS of 0.55 (standard error (SE) 0.11) in absent/low CTEN expressing tumours compared with 0.42 (SE 0.05) in the moderate/high CTEN expressing group. 

Notably, however, when patients were stratified by HPV status, the significant survival association was only evident in HPV^+ve^ tumours (*n* = 146; *p* < 0.0001, Figure S7b), albeit with a non-significant trend in HPV^−ve^ tumours (*n* = 113; *p* = 0.066, Figure S7a). 

Subgroup analysis revealed significant treatment differences between HPV^+ve^ and HPV^−ve^ tumours; use of radiotherapy either as a primary or adjuvant treatment modality, was significantly more common in HPV^+ve^ disease (Table S1), so we repeated the survival analysis stratifying patients according to treatment receive either surgery or (chemo)radiotherapy as the primary treatment modality. Patients receiving neoadjuvant chemotherapy prior to surgery (*n* = 5) were excluded from analysis. In patients treated with surgery, there was no association between level of CTEN expression and survival (pooled, Figure 5c, *n* = 97, *p* = 0.172; HPV^−ve^, Figure S7c, *n* = 45, *p* = 0.770; HPV^+ve^, Figure S7e, *n* = 52, *p* = 0.337). However, in both HPV^−ve^ and HPV^+ve^ patients treated with (chemo) radiotherapy, CTEN expression was associated with poor survival (pooled, Figure 5d, *n* = 141, *p* < 0.0001; HPV^−ve^, Figure S7d, *n* = 55, *p* = 0.021; HPV^+ve^ cohorts, Figure S7f, *n* = 86, *p* = 0.001). The negative association between CTEN and overall survival (OS) as well as 3-year disease-specific survival (DSS) was maintained when analysing all patients undergoing organ preservation treatment, separated by radiotherapy (RT) or concurrent chemoradiotherapy (CRT; log-rank 0.003 and <0.001 for RT and CRT 3-year OS, respectively; log-rank 0.04 and 0.031 for RT and CRT 3-year DSS, respectively). On univariate analysis, moderate/high CTEN expression was associated with a reduced OS (HR 2.94, 95% CI 1.97–4.38, *p* < 0.0001), along with HPV^−ve^ status (HR 2.72, 95% CI 1.88–3.94, *p* < 0.0001), current smoker (HR 1.78, 95% CI 1.16–2.72, *p* = 0,01) and advanced primary tumour (T3/4) stage (HR 2.36, 95% CI 1.63–3.44, *p* < 0.0001; Table S2). A multivariate model was tested utilising a stepwise backward likelihood ratio method, starting with the above significant variables on univariate analysis (CTEN expression, HPV status, smoking status and T stage) as well as all CTEN expression interaction terms. All interaction effects were non-significant and removed from the model, resulting in a model retaining CTEN as a significant variable (HR 4.48, 95%CI 1.98–10.12, *p* < 0.0001).

For those undergoing RT (*n* = 52), there was a positive correlation between moderate/high CTEN expression and disease-related death at 3 years (Chi-squared 7.139, *p* = 0.008) and a positive likelihood ratio (LR) and sensitivity of 1.56 and 0.947, respectively. In those patients undergoing concurrent CRT (*n* = 89), the chi squared and P value, positive LR and sensitivity are 3.894, 0.048, 1.53 and 0.765, respectively. These data suggest that CTEN expression may have utility as a predictive marker for radiotherapy/CRT resistance.

## 3. Discussion

There is growing evidence that CTEN expression supports tumour progression [22,23,24,27,45,46,47], associated, in the majority of cases, with advanced stage and aggressive disease [23,24,25,26,45,46,47,48,49]. Katz et al.’s [22] seminal paper demonstrating a CTEN-induced motile phenotype through extracellular matrix (ECM) disruption in an epidermal growth factor (EGF) driven mammary cell model has formed the basis of many of the recent hypotheses on the pro-oncogenic effects behind this protein. However, EGF is not the only growth factor involved in the regulation of Tensin expression and signalling. Other factors and cytokines, including mesenchymal epithelial transition (MET) and extracellular signal-related (ERK) kinase, fibroblast growth factor (FGF2), nerve growth factor (NGF), insulin-like growth factor (IGF-1), transforming growth factor β (TGF-β) and interleukins 6 and 13 (IL-6, IL-13), have also been implicated, suggesting that CTEN may represent a nodal convergence of multiple signalling pathways [50,51,52]. The numerous and varied proposed CTEN-signalling pathways across studies and cell lines suggest diverse roles in different tissue types and support an organ/system-based focus for further investigation. Here, we report, for the first time, its clinical and functional significance in head and neck malignancy. 

Online analysis of publicly available databases consistently showed CTEN overexpression in HNSCC tissue, validated across multiple HNSCC datasets and also other cancer types. Furthermore, the pattern of expression supported its involvement in more advanced tumour progression, more commonly upregulated in HPV^−ve^ tumours, clinically recognised as a more aggressive disease subtype with significantly reduced prognosis compared to HPV^+ve^ disease. However, no functional role for CTEN in HNSCC is yet described, and while CTEN has been shown to promote distant metastasis in colorectal cancer [26], this is not a clinical feature typically associated with HNSCC. The impact of CTEN expression on locoregional disease was, therefore, an important area for investigation in HNSCC, and the generation of stable knockdown cell lines allowed us to explore the contribution of CTEN to disease progression in a relevant orthotopic model which resembled human disease both morphologically and in its locoregional pattern of spread. 

Cell invasion plays a major role in tumour progression, and previously published observations that Tensins localise to focal adhesion complexes and interact with integrin cytoplasmic domains [22] have implicated these proteins as potential regulators of tumour cell motility and interactions with the ECM. Our in vitro data demonstrated that CTEN has an important role in promoting cell motility and invasion. However, we also found that the function of CTEN extends beyond this and present novel findings of a link between CTEN expression and suppression of apoptosis. It is possible that CTEN may affect apoptosis through regulating integrin-dependent interactions with the ECM; apoptosis can be stimulated by disruption of cell adhesion and spreading, implicating a critical role for focal adhesions in cell survival. Lo and colleagues have previously demonstrated an association between caspase-3 and CTEN, with the subsequent CTEN-cleaved fragments significantly affecting cellular growth [53]. 

Recent work also suggests potential links between these pleiotropic effects; Ilyas and colleagues identified a CTEN-mediated post-transcriptional stabilisation of the transcription factor Snail, promoting epithelial-to-mesenchymal transition (EMT) and generating a motile phenotype [54]. As well as its established role as an EMT transcription factor, Snail additionally functions to suppress apoptosis [55], thus we can hypothesise the existence of a CTEN-SNAIL signalling axis which acts to both promote invasion and suppress apoptosis, promoting tumour cancer cell dissemination and survival. 

Of particular interest was our retrospective analysis of the potential clinical significance of CTEN expression in HNSCC. Caspases are key regulators of the apoptosis pathway [56], and whether the process is initiated by the intrinsic or the extrinsic pathway, both pathways converge upon the common execution phase, comprising a series of caspase reactions [57] in which caspase-3 is the key effector. Ionising radiation-induced cell deaths occur via several mechanisms [58,59,60] but those resulting from apoptosis are recognised to be mediated by caspase-3 [61], mediated by both p53-dependent and -independent pathways [62,63]. The p53-dependent pathway is particularly important in cells with high p53 mRNA expression, such as HNSCC, where updated TCGA analysis of 510 cases demonstrates that up to 70.4% of tumours have *TP53* mutations [64]. Inhibition of caspase-3 has also been shown to protect from radiation-induced apoptosis [65,66]. HNSCC are thus susceptible to radiation-induced apoptosis [67]. Understanding the relationship between radiotherapy and apoptotic pathways is vital for the identification and study of novel radiosensitisation markers, targets and therapeutic opportunities. Alerted to the strong association in our cohort between CTEN and survival in a HPV^+ve^ HNSCC group, in which organ preservation modalities comprised the larger treatment arm, we confirmed that expression of CTEN appears to regulate a number of key apoptotic markers. The similar effect across both HPV^+ve^ and HPV^−ve^ tumour subtypes was notable given the molecular differences in these tumour subtypes, supporting an extrinsically activated pathway, i.e., radiation-mediated cell death, as the mechanism behind this effect. Although CTEN demonstrated an overall prognostic effect in our dataset, subgroup analysis showed that this was limited to (HPV−ve and HPV+ve) patients treated with radiotherapy/CRT. This suggests that CTEN may have utility as a predictive marker for radioresistance. With a demonstrable effect on the apoptotic pathway to explain this effect and a simple pathological test to use in practice, the high sensitivity and positive likelihood ratio of moderate/high CTEN expression to predict treatment response warrant further investigation in a prospective trial. 

In summary, we show that CTEN is commonly upregulated in both HPV^−ve^ and HPV^+ve^ tumours and has pleiotropic effects, promoting tumour cell invasion and suppressing apoptosis. The latter function may pay an important role in conferring HNSCC resistance to radiotherapy and, as such, may be a useful predictive biomarker and represent a potential therapeutic target as a radiosensitiser. 

## 4. Materials and Methods

### 4.1. Antibodies and Reagents

The monoclonal antibodies (mAbs) used were commercially obtained: anti-human CTEN MAB6925 (R&D Systems, MN, USA), pancytokeratin (AE1/AE3; Dako, Santa Clara, CA, USA) and anti-cleaved caspase 3 (ab2302; Abcam, Cambridgeshire, UK). Rat-tail type I collagen and MatrigelTM were from BD Biosciences (Oxford, UK). 

### 4.2. Cell lines and Culture

HNSCC-derived HPV^−ve^ cell lines SCC-25 [68] and UM-SCC89 [69] were cultured in a standard medium consisting of Dulbecco’s modified Eagle’s medium (DMEM; Sigma Aldrich, St. Louis, MO, USA):Ham’s F12 (1:1; Lonza, Basel, Switzerland), containing 10% (*v⁄v*) foetal bovine serum (FBS) and 2mM L-glutamine. Two immortalised cell lines derived from the upper aerodigestive tract from suspected HPV^+ve^ tumours, UD-SCC-2 and UPCI: SCC90 [70,71], were kindly supplied by Susanne M. Gollin (University of Pittsburgh, USA; shortened to SCC2 and SCC90, respectively, in Results). These cell lines, along with Detroit 562 (metastatic primary oropharyngeal SCC [72]) and MCF7 (breast adenocarcinoma [73]) were cultured in 10% FBS-supplemented DMEM. Further, the cell lines H357 (a primary oral tongue SCC [74]), 5PT (a cisplatin resistant supraglottic SCC [75]) and VB6 (H357 transfected with α_v_ and β_6_ cDNA [76]) were cultured in a standard keratinocyte growth medium (KGM): α-modified Eagle’s medium (α-MEM) containing 10% foetal bovine serum (FBS; Globepharm, Surrey, UK), supplemented with 1.8 × 10^−4^ M adenine, 5 mg/mL insulin, 0.5 mg/mL hydrocortisone and 10 ng/mL epidermal growth factor (Sigma Aldrich). All cells were tested routinely for mycoplasma throughout experimentation. Cell counting for all functional assays was performed utilising a CASY counter (Roche, Basel, Switzerland). 

### 4.3. Gene Silencing

Cells were transfected with validated, and optimised siRNA oligonucleotides at 30 nM final concentration against CTEN (Cat#: 4392421; Life Technologies, Carlsbad, CA, USA) or silencer negative control siRNA (Cat#: AM4635; Applied Biosystems, Foster City, CA, USA) using Oligofectamine™ transfection reagent according to the manufacturer’s protocol (Invitrogen, Carlsbad, CA, USA). 

For stable CTEN depletion, HuSH 29mer GFP-tagged shRNA plasmid constructs against CTEN in lentiviral particles, together with a scrambled control, were commercially obtained from Origene (Cat#:TL300896V; Rockville, MD, USA). SCC-25 cells were transfected according to the manufacturer’s protocol at a target 50% confluence with virus particle mixture at a multiplicity of infection (MOI) of 2, together with 8 µg/mL Polybrene (Santa Cruz Biotechnology, Dallas, TX, USA). Cell lines with stable incorporation of the plasmid DNA were isolated by puromycin (Sigma-Aldrich, St. Louis, MO, USA) at 2 µg/mL [77]. Cell clone populations with stably significant reduced CTEN expression were screened by GFP expression and then selected by Western blotting (Figure S8a,b). 

Functional assays were performed at 24 (wound scratch, colony forming and organotypic assays) or 48 h (Transwell^®^ invasion assays) post-transfection following confirmation of knockdown effect duration (with knockdown confirmation). 

### 4.4. Polymerase Chain Reaction (PCR)

RNA was extracted from samples to be analysed using an RNAeasy^®^ Kit (Qiagen, Hilden Germany) and cDNA was synthesised using qScript^®^ cDNA SuperMix (Quanta Biosciences, VWR, Beverly, MA, USA) according to the manufacturer’s instructions. RT-qPCR was performed with PerfeCTa^®^ FastMix II (Quanta Biosciences, VWR), using the commercially available FAM-labelled TaqMan Gene Expression Assay for CTEN (Hs00262662_m1; Applied Biosystems, CA, USA) and using the comparative CT method, normalised to human *ACTB* endogenous control (4326315E, Thermofisher, MA, USA).

### 4.5. Western Blot Analysis

Cells were lysed in NP40 buffer (Biosource, Invitrogen, Paisley, UK). Samples containing equal amounts of protein were electrophoresed under reducing conditions in 8–10% SDS-PAGE gels. Protein was electroblotted to polyvinylidene difluoride (PVDF) membranes (Amersham Biosciences, Buckinghamshire, UK). Blots were probed against primary antibodies of interest and horseradish peroxidase-conjugated anti-rat or anti-mouse (Dako) were used as secondary antibodies. Bound antibodies were detected with the enhanced chemiluminescence Western blotting detection kit system (Amersham). Blots were probed for HSC70 (Santa Cruz Biotechnology) as a loading control. 

### 4.6. Database Analysis

The mRNA expression of CTEN in HNSCC was investigated inside the Oncomine 4.5 database, one of the largest freely accessible data mining platforms available online [33], drawing on two of the largest available dedicated head and neck series outside of The Cancer Genome Atlas (TCGA), namely Ginos HNSCC [35] and Peng HNSCC [34]. CTEN expression in head and neck tumour samples were compared to correlative normal tissue and *p* < 0.05 utilised as a statistically significant cutoff. 

The TCGA data were also interrogated utilising two open-access online interfaces that enable analysis and visualisation of transcriptomic and expression data: UALCAN [78] and c-BioPortal [42]. The former allowed a global overview of expression data between HNSCC specimens (*n* = 520) and matched normal tissue (*n* = 44), whereas the latter allowed a more in-depth analysis of mRNA expression by tumour stage and HPV status. 

### 4.7. Cell Invasion Assay

Cell invasion was analysed using Transwell^®^ assays (8 μm pore size, polycarbonate membrane, Corning^®^ Costar^®^, Wiesbaden, Germany) as previously described [79]. Briefly, a layer of Matrigel^TM^ (BD Biosciences, San Diego, CA, USA) diluted 1:2 with DMEM was placed on top of each insert prior to seeding 5 × 10^5^ cells/200 µL in each chamber. A 72-h incubation at 37 °C was performed to allow invasion of cells into the lower chamber and counted using a CASY automated counter.

### 4.8. Scratch Wound Assay

Cells were plated at 8 × 10^5^/well in a 12-well plate and allowed to adhere to form a confluent monolayer overnight. The next day, a scratch along the centre of the well was introduced with a sterile P200 pipette tip and ruler guide. Detached cells were removed, and serum-free medium was added. For time-lapse capture, the plates were transferred to a humidified 5% CO_2_ chamber at 37 °C for 48 h, with images of cells in phase acquired every 5 min using a microscope (IX81, Olympus) controlled by cell^P software, at 20× objective of a Zeiss AxioCam MRm camera, together with automatic focussing. Collected images were processed using ImageJ software [37].

### 4.9. Colony Forming Assay

SCC-25 cells following either negative control silencer siRNA or CTEN siRNA transfection were seeded in 6-well plates at 2000 cells/well. Standard growth media were utilised for both cell populations. At completion (10 d), cells were fixed in a 3% crystal violet/10% formalin solution. Following collation of scanned images, the ‘ColonyArea’ plugin of Image J [80] was utilised, allowing automated thresholding and analysis, to calculate both % area and intensity of colonies.

### 4.10. 3D Cell Proliferation Assay

In addition to standard cell counts performed on a 24-well plate, a 48-h cell monolayer culture and prolonged assays to count proliferation of cells suspended in a collagen gel were performed. Briefly, collagen gels were prepared on ice, comprising 7 volumes of rat tail collagen type I, 1 volume of 10× DMEM, 1 volume of serum and 1 volume of SCC-25 cells (control/*CTEN* siRNA transfected or shRNA transduced), suspended at a final concentration of 5 × 10^4^ cells/mL. The mixture was neutralised by addition of 0.1M sodium hydroxide with gentle mixing. Then, 1 ml of this mixture was pipetted into each well of a 24-well plate and allowed to polymerise for 1 h at 37 °C. Subsequently, 1mL of 10% DMEM was added on the top of each gel to prevent dehydration and incubated at 10% CO_2_ and 37 °C for 6 d. At each desired time point for proliferation count, media were aspirated from the control- and knockdown cell-containing gels and replaced with 10% collagenase (Sigma-Aldrich). The gels were placed on a 37 °C heating plate and left on a plate shaker for 1.5 h until complete gel digestion had occurred. Cell counts were then determined using the automated CASY counting system.

### 4.11. Organotypic Culture

Organotypic cultures were prepared as previously described [79]. A 1 ml mixture comprising 3.5 volumes type I rat tail collagen (Merck Millipore), 3.5 volumes Matrigel^TM^ (Becton-Dickinson), 1 volume 10× DMEM, 1 volume foetal calf serum and 1 volume of 10% DMEM together with 2.5 × 10^5^/mL HFFF2 cells was allowed to polymerise. A mixture of transient or stable CTEN knockdown HNSCC cells (5 × 10^5^) and HFFF2 fibroblasts (25 × 10^4^) in a combined volume of 1mL of DMEM was then added dropwise onto the top of the gel. After 24 h incubation, the gels were raised onto nylon sheet-coated stainless-steel grids. After 7 d incubation at 37 °C, the gels were bisected, fixed in formal-saline and processed to paraffin. Sections (4 μm) were stained for H&E +/− pan-cytokeratin. 

### 4.12. In Vivo Orthotopic Head and Neck Model 

Male athymic nude Ncr*^−nu/nu^* mice, 6–8 weeks of age, were purchased from Charles River (UK) and housed in a pathogen-free animal facility. All animal procedures were performed under the UK Home Office Project licence no. P8969333C in accordance with a previously published protocol for oral orthotopic models [81] and were approved by an institutional review board. GFP-tagged stable CTEN knockdown SCC-25 cell lines and their transduced controls were used, and following collection and counting, 50,000 cells/30 µL additive-free DMEM were prepared. Mice were put into two groups of 10 for control or CTEN-knockdown cell injection. All of the mice were anaesthetised with an inhalational agent (isoflurane/O_2_ mix) and underwent submucosal tongue injection of the allocated cell line directly into the anterior tongue using a 100-µL Hamilton syringe (Hamilton Co.) with a 30-gauge hypodermic needle. Mice were examined once weekly for the development of tongue tumours and sacrificed when they had lost >20% of their pre-injection body weight. A soft diet was instigated once any tumour was visible. Following termination, mice were imaged on fluorescence imager (IVIS, Perkin Elmer) to identify those with potential cervical metastasis and help direct dissection. All tongue tumours and pathological cervical lymph node specimens were then processed for histopathological examination. Tumour volume (V) was calculated using the volume formula: (V = ½ (L × W^2^)), where L = length and W = width [82].

### 4.13. Apoptosis Protein Array

Analysis of protein expression by the Proteome Profiler Human Apoptosis Array Kit (R&D Systems) was performed according to the manufacturer’s instructions. Briefly, extracted protein samples from stable CTEN-depleted SCC-25 cells and appropriate matched controls were incubated with each array at 4 °C overnight on a rocking platform shaker. Any unbound protein was then removed and membrane incubation with the primary antibody and secondary antibody proceeded, with appropriate washes as directed. Protein spots were visualised using the chemiluminescence reagents commercially supplied. The intensity score of each duplicated array spot was measured with ImageJ software, and the average intensity was calculated as a ratio to the control spots at the corner of each membrane. The identity and map of all the antibodies on the arrays can be found in Table S3 and Figure S10.

### 4.14. RNA Sequencing and Data Analysis

RNA was extracted at separate time points from transfected cell populations to include both CTEN knockdown cells, confirmed by PCR, and paired control siRNA cells, utilising the RNAeasy Kit (Qiagen, Hilden, Germany) according to the manufacturer’s instructions. Resulting RNA was re-suspended in RNAse-free water and quantified on a spectrophotometer and RNA quality was determined using Bioanalyser analysis (Agilent Technologies Inc., Santa Clara CA, USA; Figure S9) to obtain RNA integrity numbers prior to downstream processing. For commercial analysis, 250–300 ng of total RNA at a minimum concentration of 25 ng/µL was sent to Expression Analysis Genomic Services (Durham, USA). RNA sequencing was performed using the Illumina Truseq Stranded protocol with paired-end sequencing and 20 million reads per sample. Reads were mapped to the human genome (hg19) with Tophat 2.0.13 [83], indexed and sorted with Samtools −1.2 [84] and counted using HTSeq [85] to allow normalisation and differential gene expression analysis. 

Gene ontology (GO) terms associated with biological processes and biological pathways that were significantly over-represented for differentially expressed genes (DEGs) (*q*-value < 0.05) were identified with the ToppGene web tool [86] and functional enrichment analysis was performed using ToppFun from the ToppGene suite. Statistical significance of different GO terms and pathways were estimated using hypergeometric testing with false discovery rate (FDR) adjustment for multiple testing using the Benjamini and Hochberg method [87]. GO terms and functional pathways with FDR-corrected *p*-value < 0.05 were considered significant. Significant GO terms were visualised in semantic similarity-based scatterplots generated by REVIGO [44].

### 4.15. Tissue Microarry Array (TMA) Production and Immunohistochemistry

Immunohistochemical analysis was performed on a tissue microarray of consecutively-treated patients with oropharyngeal squamous cell carcinoma (OPSCC; *n* = 259) from University Hospital Southampton (2000–10), Poole NHS Foundation (2000–6) and Barts and the London NHS Trust (2000–2006), as described previously [16] (Research Ethics Committee [REC] references 09/H0501/90 and 07/Q0405/1). Tumours classified as HPV^+ve^ were positive for both p16 immunohistochemistry and HPV ISH. Pathological features were recorded as per the AJCC TNM 7th edition. Immunochemistry was performed using antibody to CTEN (R&D Systems, MN) at a 1:200 concentration with a high pH (Tris-EDTA buffer (pH 9.0)) antigen retrieval protocol. Subsequent slides were scored by two blinded investigators (K.M./J.F.) as high (moderate/high strength staining) or low (absent/low strength staining), consistent with prior CTEN literature [26,27]. Patient demographics can be seen in Table 1. 

### 4.16. Statistical Analysis

For comparisons between experimental groups in functional assays, a Student’s *t*-test was used. For other datasets where normal distribution was not evident, non-parametric data analysis was performed (Mann–Whitney test for unpaired, Wilcoxen matched-pairs signed rank test; Prism v6 for Mac, Graphpad Software, San Diego, USA). Figures show representative examples of independent repeats (*n* = 3 unless otherwise stated in figure legends), with error bars representing standard deviation (SD). For TMA/database analysis, SPSS Statistics (v22 for Mac, IBM, NY, USA) was used.

The primary endpoint for survival analysis was death from any cause or overall survival (OS). Survival time was measured from the date of diagnosis until date of death from any cause or date last seen alive. Kaplan–Meier survival curves were produced from our OPSCC database using clinicopathological patient data (available in *n* = 259). Death from other causes or loss to follow-up was marked as censored for analysis. In addition to log-rank tests, univariate Cox proportional hazard regression was used to test the association of OS with other potential covariates. Statistically significant covariates were used to develop a multivariate Cox proportional hazards regression model to assess the independent prognostic significance of CTEN. For all analyses, a *p* value of equal to or less than 0.05 was considered to be statistically significant. 

## 5. Conclusions

We have demonstrated CTEN to be a highly expressed, clinically relevant marker in aggressive HNSCC disease. It modulates several functional effects both in vitro and in vivo to support tumour growth, promoting tumour invasion and cancer cell survival. A potential role in modulating apoptotic response to therapy suggests utility as a predictive biomarker for radiotherapy treatment response or as a radio-sensitising target in malignant disease.

## Figures and Tables

**Table 1:** Comprehensive oropharyngeal squamous cell carcinoma (OPSCC) database demographics, according to the American Joint Committee on Cancer (AJCC) TNM 7th edition.

Category	All OPSCC		HPV-Positive OPSCC		HPV-Negative OPSCC	
	Frequency	*%*	Frequency	*%*	Frequency	*%*
**Final HPV Status**						
Negative	113	43.6	-	-	-	-
Positive	146	56.4	-	-	-	-
**Gender**						
Female	67	25.9	36	24.7	31	27.4
Male	192	74.1	110	75.3	82	72.6
**Age at Diagnosis**						
<50	54	20.8	39	26.7	15	13.3
50–69	156	60.2	88	60.3	68	60.2
70+	49	18.9	19	13.0	30	26.5
**Smoking**						
Non-smoker/Ex-smoker	102	48.6	78	62.9	24	27.9
Current smoker	108	51.4	46	37.1	62	72.1
**Alcohol**						
Non-drinker/Ex drinker	30	15.5	18	16.1	12	14.8
Current drinker	163	84.5	94	83.9	69	85.2
**Overall Stage**						
I/II	45	17.5	11	7.6	34	30.4
III/IV	212	82.5	134	92.4	78	69.6
**T Stage**						
Tis/T1/T2	156	64.2	99	69.2	57	57
T3/T4	86	35.4	43	30.1	43	43
Tx	1	0.4	1	0.7	0	0
**Nodal Metastases**						
No	51	21.1	12	8.5	39	39
Yes	191	78.9	130	91.5	61	61
**N Stage**						
N0–N2a	81	33.5	33	23.2	48	48
N2b–N3	161	66.5	109	76.8	52	52
**Distant Mets at Presentation**						
No	239	98.8	141	99.3	98	98
Yes	3	1.2	1	0.7	2	2
**Tumour Grade**						
Well/moderately differentiated	88	34	29	19.9	59	52.2
Poorly differentiated	171	66	117	80.1	54	47.8
**Primary Treatment**						
Surgery	97	37.5	52	35.6	45	33.1
Neoadjuvant chemotherapy, surgery	5	1.9	4	2.7	1	1.7
Radiotherapy	52	20.1	22	15.1	30	17.8
Chemoradiotherapy	89	34.4	64	43.8	25	30.4
None/Palliative	16	6.2	4	2.7	12	5.5
**Surgical Treatment Breakdown**						
Surgery only	20	20.8	4	7.7	16	35.6
Surgery, PORT	68	70.8	41	78.8	28	62.2
Surgery, POCRT	8	8.3	7	13.5	1	2.2
**Margin Status**						
Negative	61	61	29	60.4	28	60.9
Close	21	21	11	22.9	9	19.6
Positive	18	18	8	16.7	9	19.6
**CTEN Score**						
Absent/low	115	44.4	86	58.9	29	25.7
Moderate/high	144	55.6	60	41.1	84	74.3

Clinicopathological features of OPSCC patients (*n* = 260), collected from University Hospital Southampton (2000–10), Poole NHS Foundation (2000–6) and Barts and the London NHS Trust (2000–6). Frequencies along with valid % for each categorical variable (bold) are listed for the total patient cohort and categorized by human papillomavirus (HPV) status. Survival data are missing for one patient from this dataset. Tis = carcinoma in situ; PORT = post-operative radiotherapy; POCRT = post-operative chemoradiotherapy.

## Supplementary Materials

The following are available online at https://www.mdpi.com/2072-6694/12/10/2963/s1, Figure S1: RNA-seq CTEN (TNS4) gene expression quantified in pan-cancer TCGA database, Figure S2: Expression and localisation of CTEN in a range of human cancer cell lines, Figure S3: Western blot anti-CTEN, Figure S4: No short-term effect of CTEN on cell proliferation, Figure S5: Effect of CTEN knockdown on activated caspase-3, Figure S6: mRNA expression correlation plots for CTEN and apoptosis markers, Figure S7: Survival analysis for oropharyngeal squamous cell carcinoma (OPSCC) subgroups, Figure S8: CTEN stable knockdown validation, Figure S9: Pre-processing and quality assessment of RNA for RNA sequencing analysis, Figure S10: Human apoptosis array coordinates map, correlating with targets listed in Table S3, Table S1: Crosstab analysis of disease and patient factors in OPSCC patient cohort, Table S2: Univariate analysis results for OPSCC patients, Table S3: Legend map for Proteome Profiler Human Apoptosis Array Kit.
