# Supplementary material for: CTEN Induces Tumour Cell Invasion and Survival and Is Prognostic in Radiotherapy-Treated Head and Neck Cancer
Source: Cancers (Basel). 2020 Oct 13;12(10):2963. doi: 10.3390/cancers12102963 (PMC7602105; doi:10.3390/cancers12102963)
Supplement: Supplementary file 1 [file cancers-12-02963-s001.pdf]

Supplementary Materials: CTEN Induces Tumour Cell Invasion and Survival and Is Prognostic in Radiotherapy-Treated Head and Neck Cancer

Jason C. Fleming, Jeongmin Woo, Karwan Moutasim, Christopher J. Hanley, Steven J. Frampton, Oliver Wood, Matthew Ward, Christopher H Woelk, Christian H. Ottensmeier, Sassan Hafizi, Dae Kim and Gareth J. Thomas

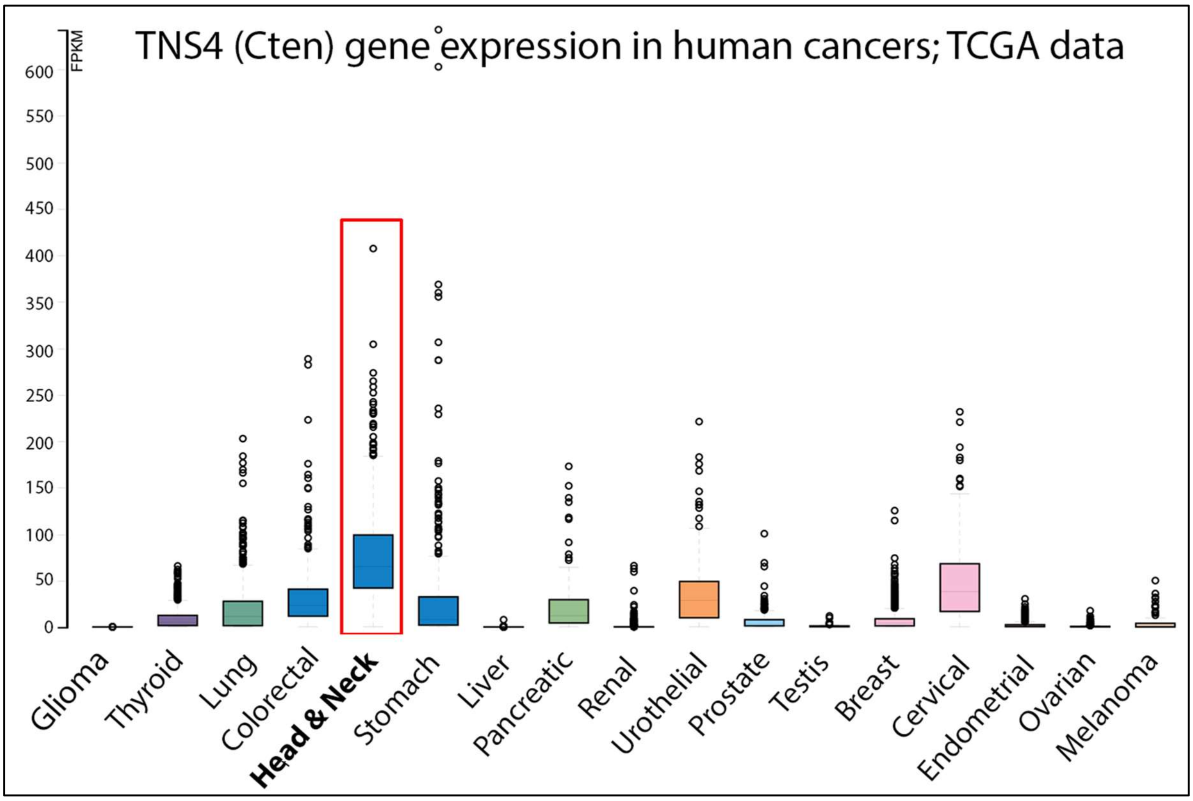

Figure S1. RNA-seq CTEN (TNS4) gene expression quantified in pan-cancer TCGA database. Red box highlights the head and neck dataset which demonstrates the highest CTEN expression across cancer types tested within the library.

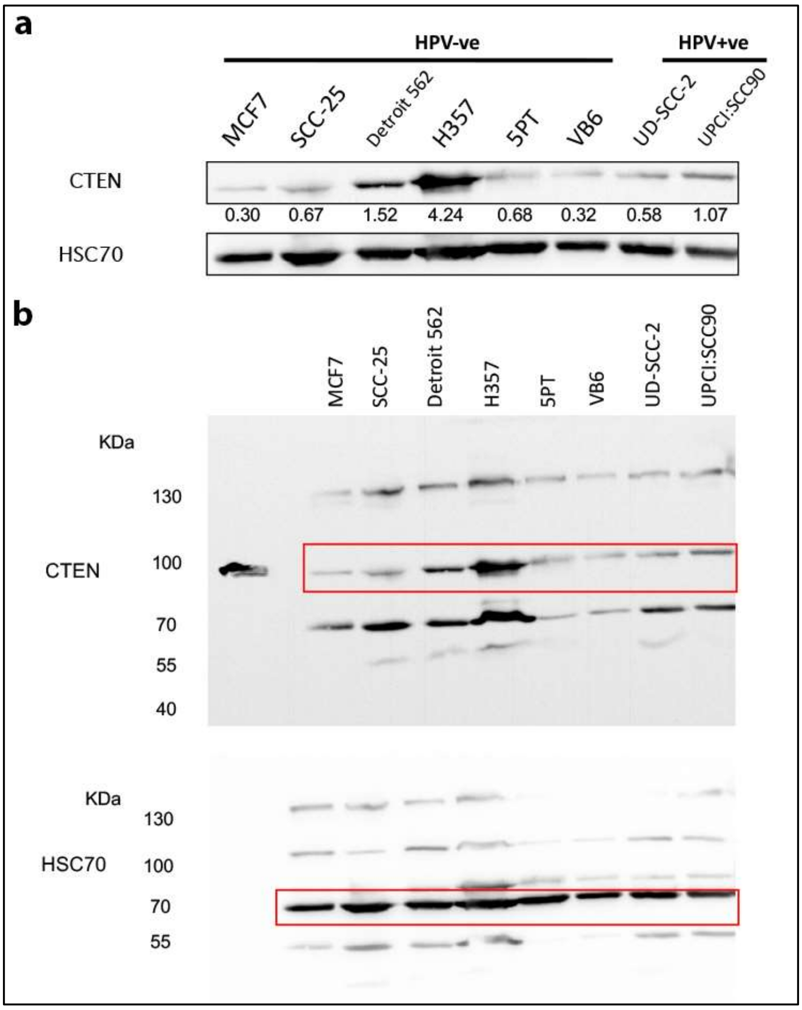

Figure S2. Expression and localisation of CTEN in a range of human cancer cell lines. (**a**) Western blot results showing CTEN expression in a range of cancer cell lines, including HPV^{-ve} (SCC-25, Detroit 562, H357, 5PT, VB6, IC6pr, C1 and BICR6) and HPV^{+ve} head and neck squamous cell carcinoma lines (UD-SCC-2 and UPCI:SCC90). Desitometry readings relative to loading control are displayed. Molecular weight markers are shown on the left. All cell lines were cultured and harvested at 80–90% confluency for lysis. A breast cancer cell line MCF7 was used as a previously published positive control [14]. HSC70 was used as loading control. (**b**) Whole western blots for CTEN (upper) and HSC70 (lower) corresponding to (**a**).

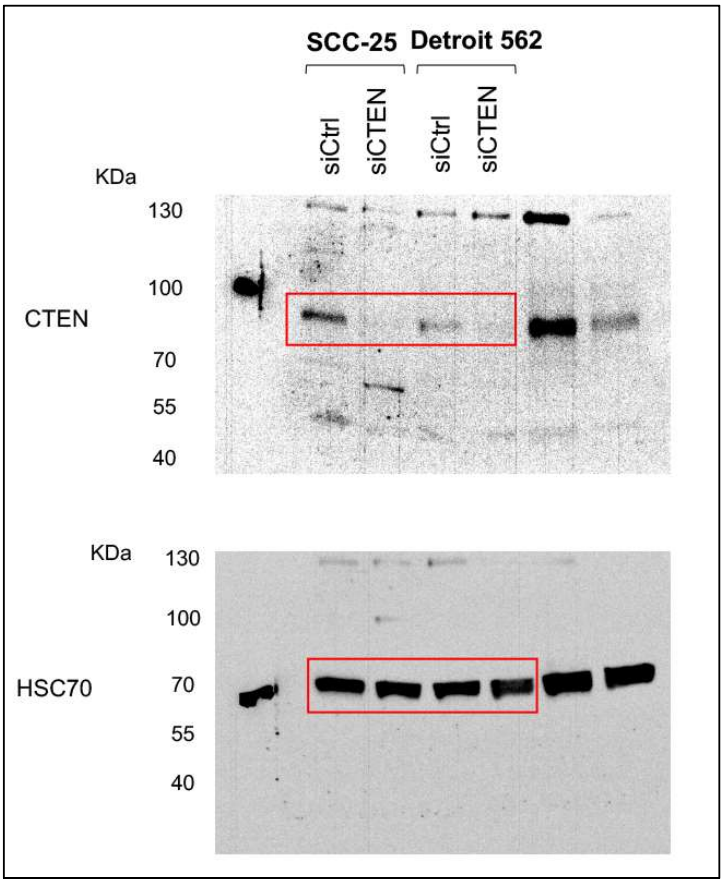

Figure S3. Western blot anti-CTEN. Whole Western blot corresponding to the presented results in Fig. 3a. Molecular weight markers are shown on the left. HSC70 is used as a loading control.

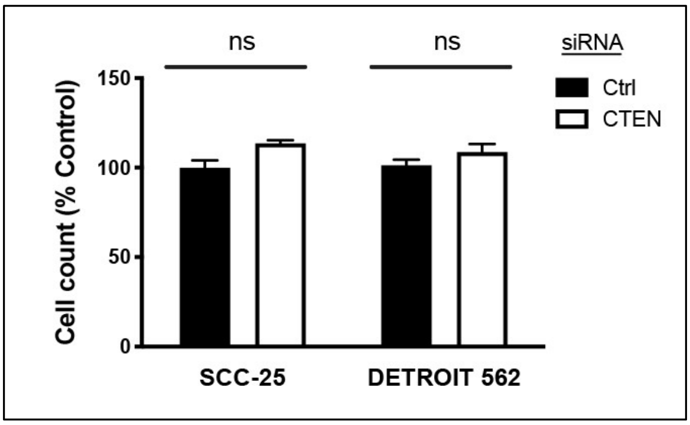

Figure S4. No short term effect of CTEN on cell proliferation**.** Two head and neck squamous cell carcinoma cell lines (SCC-25 and Detroit 562) were transfected with CTEN siRNA oligonucleotides prior to functional assessment in invasion assays (from Figure 3a). Cells that invaded through this gel towards serum-containing medium in the bottom chamber were counted after 72 h on a CASY automated cell counter. An automated proliferation assay running parallel to the functional assay was performed with the siRNA-transfected cell lines showing no significant difference between control and CTEN-knockdown cancer cell proliferation. Ns = not significant.

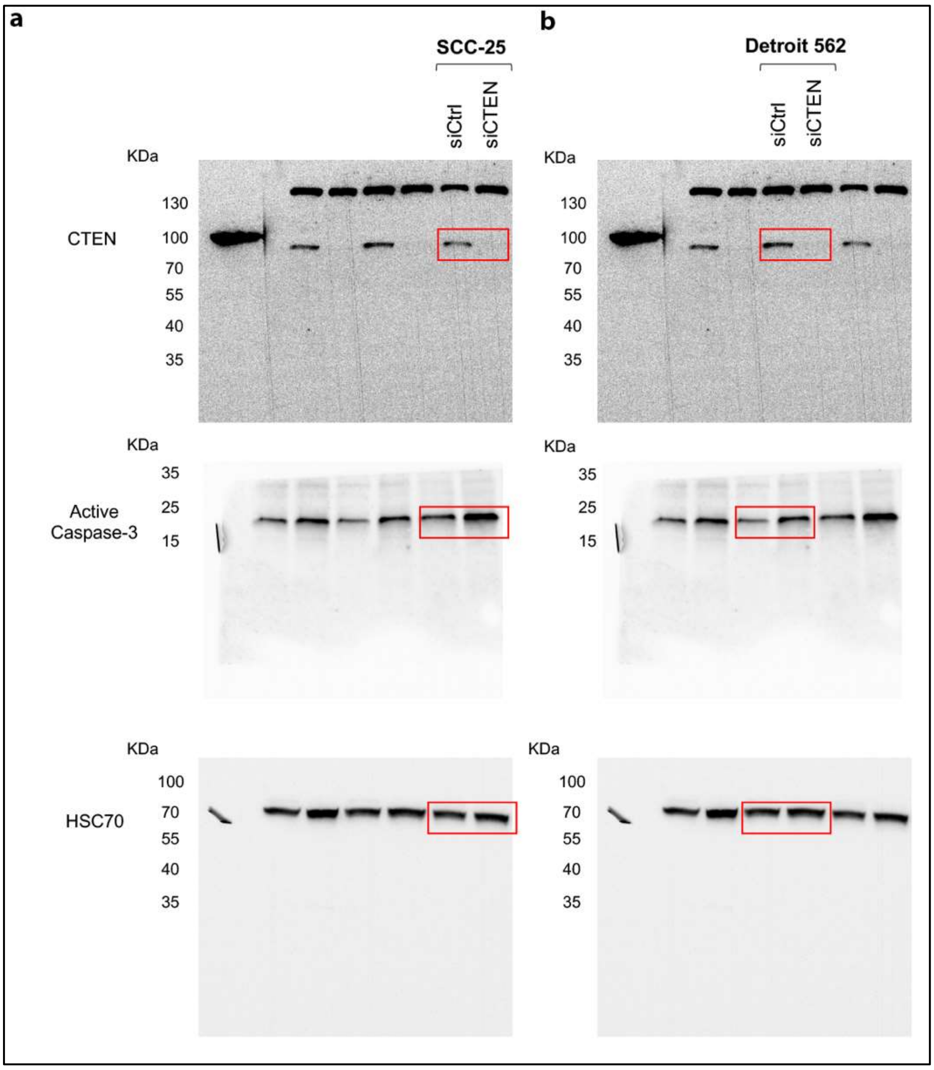

Figure S5. Effect of CTEN knockdown on activated caspase-3. Whole Western blots presented for siRNA CTEN-knockdown cells of HNSCC cell lines to examine activated caspase-3 expression in (**a**) SCC-25 and (**b**) Detroit 562. Whole Western blots for CTEN (upper), active caspase-3 (middle) and HSC70 (lower) presented corresponding to Figure 4c (lower panel).

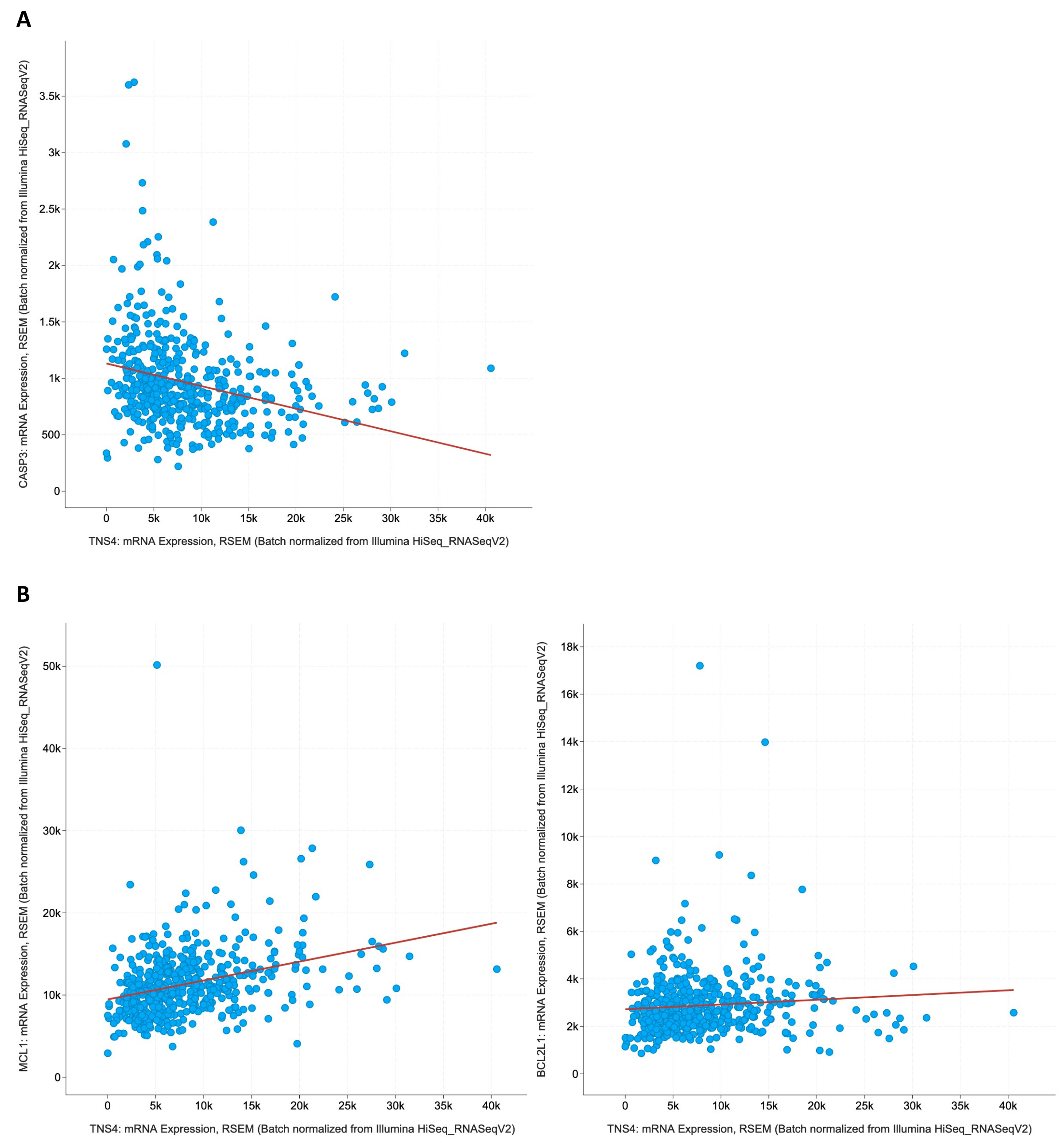

Figure S6. mRNA expression correlation plots between CTEN (TNS4; x-axis) and A) Pro-apoptotic marker CASP3 (Spearman's rho = −0.29, $p < 0.0001$) and B) Anti-apoptotic markers MCL1 (left panel, 0.37, $p < 0.0001$) and BCL2L1 (right panel, Spearman's rho = 0.15, $p = 0.0013$). Red line represents regression line of best fit.

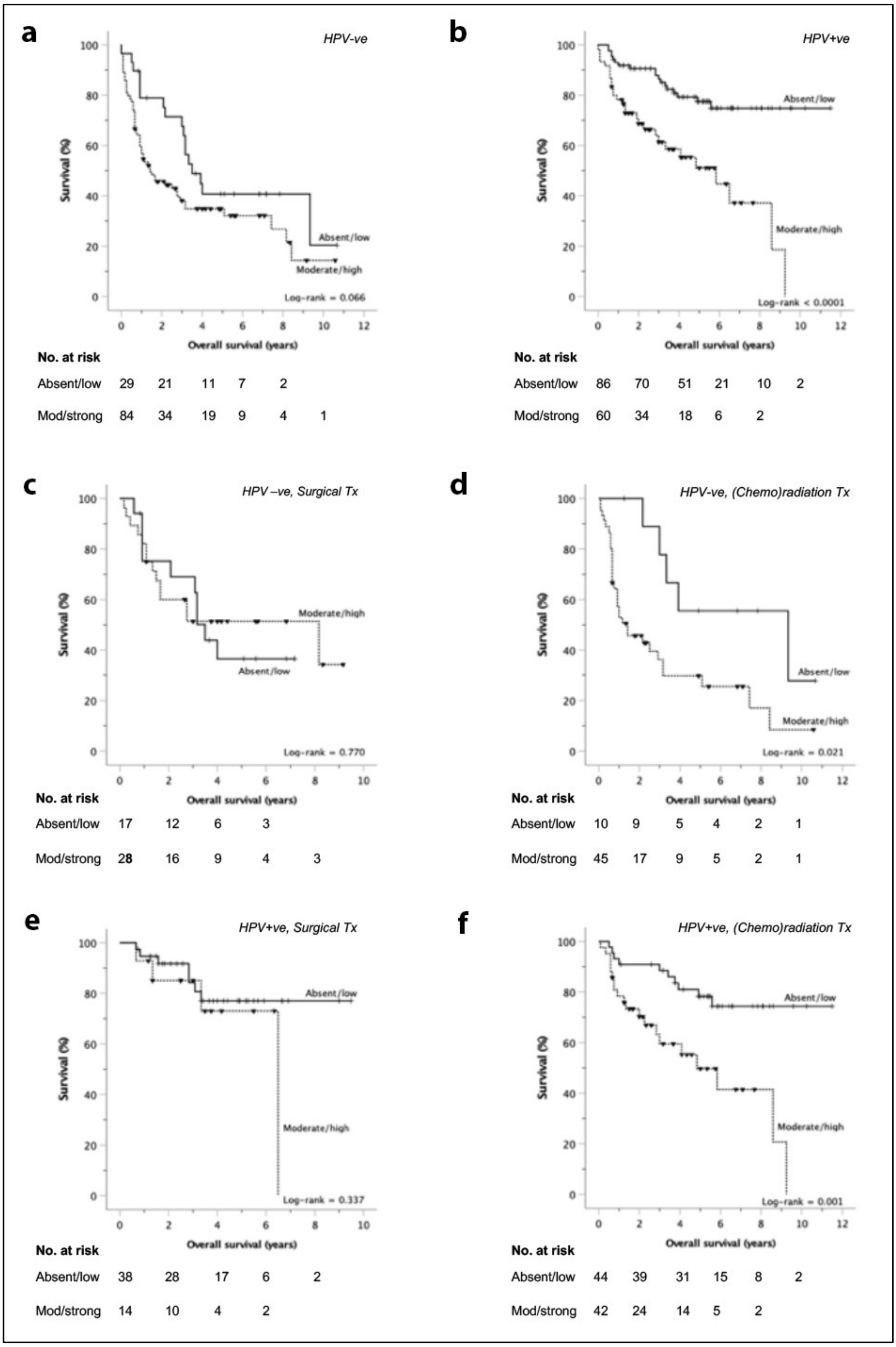

Figure S7. Survival analysis for oropharyngeal squamous cell carcinoma (OPSCC) subgroups. Kaplan-Meier overall survival curves presented for different patient cohorts, separated by CTEN expression, scored on immunohistochemistry as low (absent/weak) or high (moderate/high). No significant correlation between CTEN expression and overall survival is demonstrated for (**a**) all HPV^{-ve} patients (*n* = 113, log-rank = 0.066), (**c**) HPV^{-ve} patients treated with primary surgery (*n* = 45, log-rank = 0.770) and (**e**) HPV^{+ve} patients treated with primary surgery (*n* = 52, log-rank = 0.337). In

contrast, a significant association between high CTEN expression and reduced overall survival is evident in (**b**) HPV^{+ve} tumours (n = 146, log-rank < 0.0001), (**d**) HPV^{-ve} patients treated with primary (chemo) radiation (n = 55, long-rank = 0.021) and (**f**) HPV^{+ve} patients treated with primary (chemo) radiation (n = 86, log-rank = 0.001).

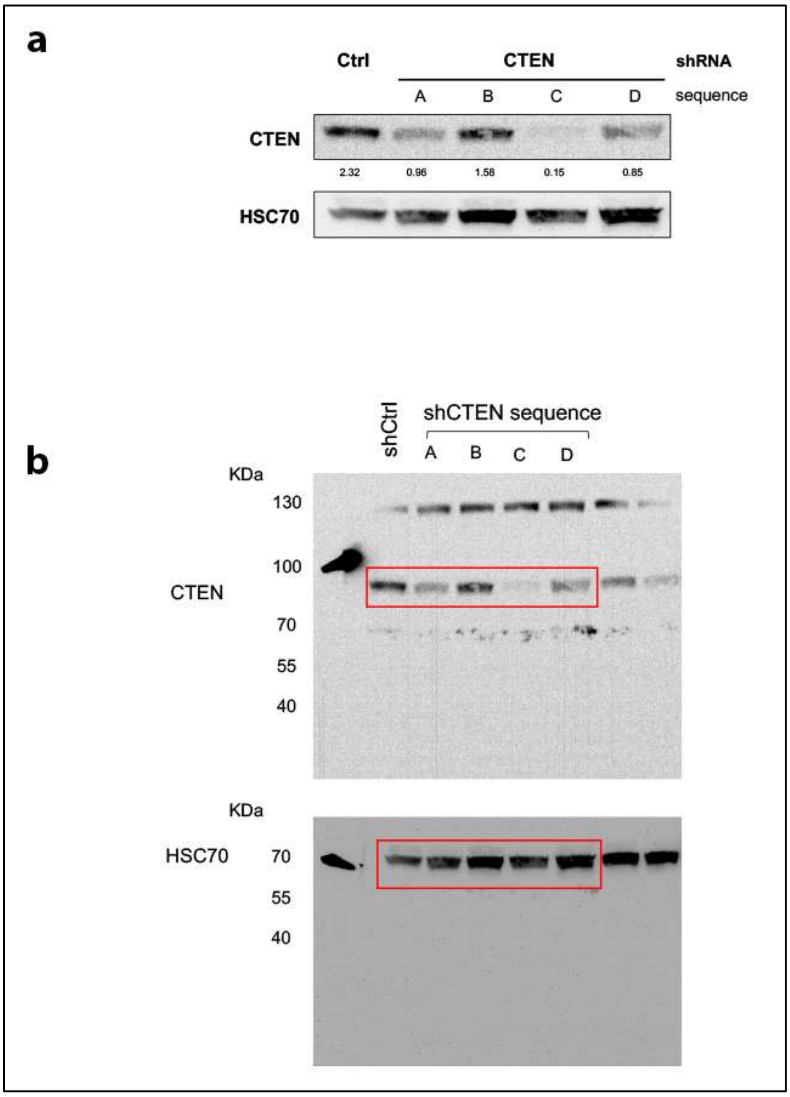

Figure S8. CTEN stable knockdown validation. (**a**) Stable CTEN-knockdown cell lines were produced using human shRNA lentiviral particles as described. Gene silencing was confirmed by examination of protein levels with Western blotting and from this result, shRNA batch C was chosen for functional experiments. Desitometry readings relative to loading control are displayed and HSC70 was used as a loading control for all Western blots. (**b**) Whole western blots for CTEN (upper) and HSC70 (lower) corresponding to (**a**).

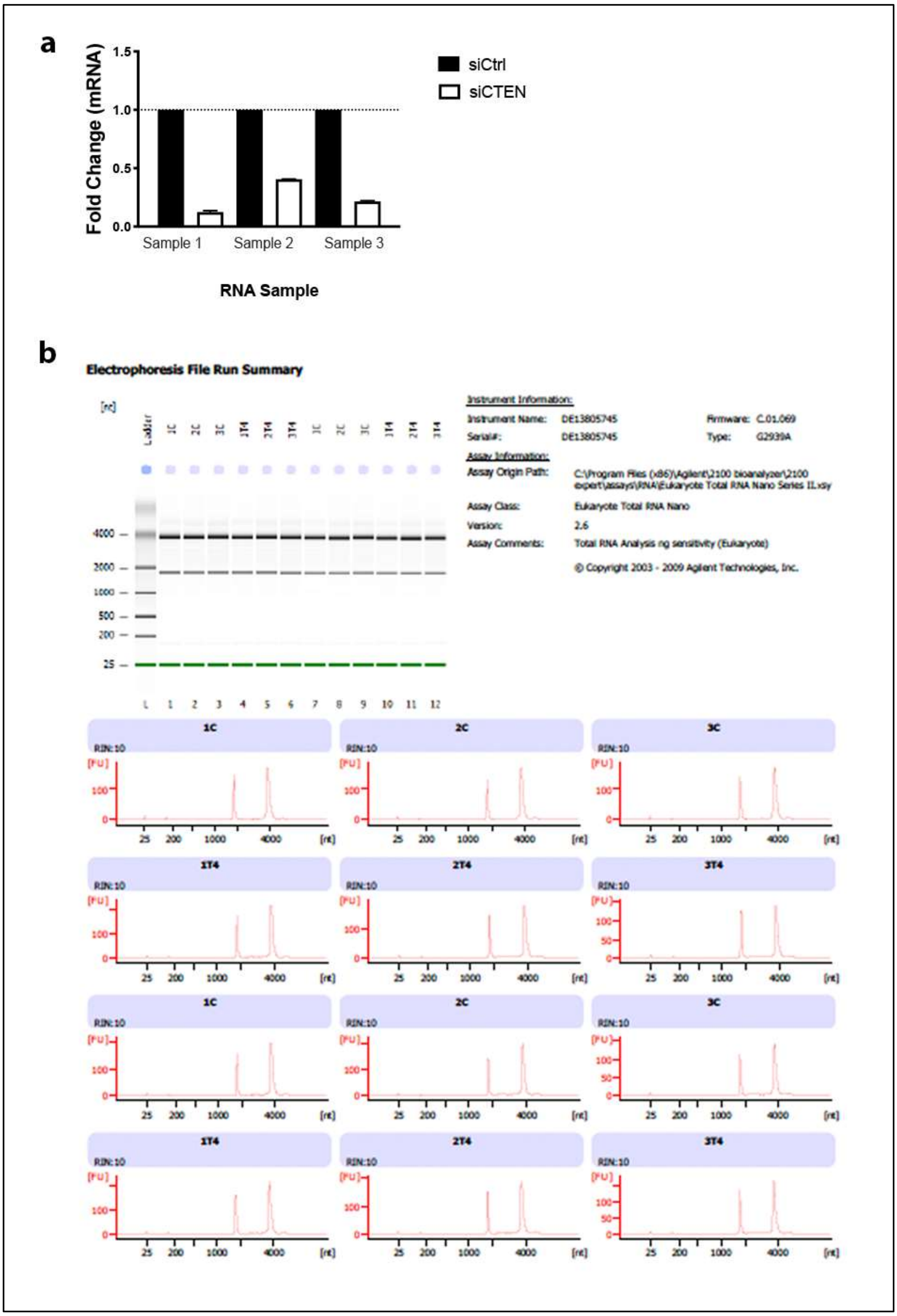

Figure S9. Pre-processing and quality assessment of RNA for RNA sequencing analysis. (**a**) Three time- and batch-independent SCC-25 cell culture populations were treated with either Ctrl siRNA or *CTEN* siRNA and collected at 48 h post-transfection. Two-step RT-qPCR was performed to confirm *CTEN* knockdown prior to performing RNA sequencing. (**b**) RNA quality was determined using Bioanalyser analysis (Agilent Technologies Inc., CA, USA) to obtain RNA integrity numbers prior to downstream processing.

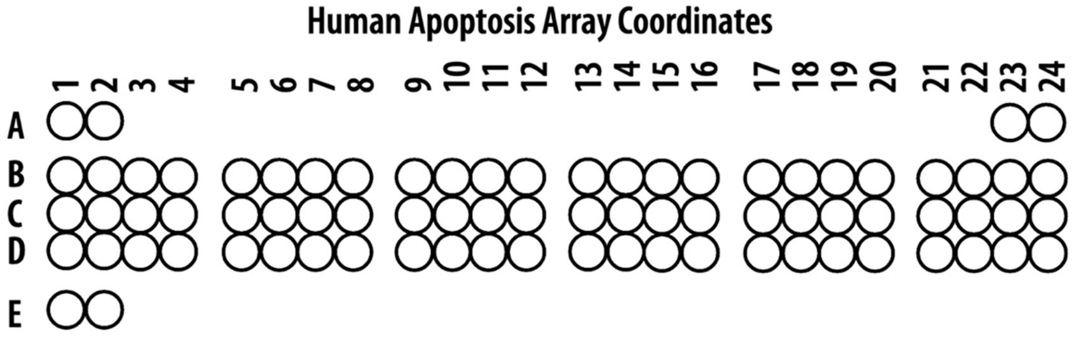

Figure S10. Human apoptosis array coordinates map, correlating with targets listed in Table S3.

Table S1. Crosstab analysis of disease and patient factors in OPSCC patient cohort (*n* = 260).

Category	HPV STATUS		χ2 Statistic	*p* Value
	HPV Positive	HPV Negative		
**T Stage**				
T1/2	99	57	4.14	**0.04**
T3/4	43	43		
**N stage**				
N0-N2a	33	48	16.16	**<0.0001**
N2b-N3	109	52		
**Disease Stage**				
Early (I/II)	11	34	22.69	**<0.0001**
Late (III/IV)	134	78		
**Smoking**				
Non/ex	78	24	24.90	**<0.0001**
Current	46	62		
**Alcohol**				
Non/Ex	18	12	0.06	0.81
Current	94	69		
**Margin**				
Negative	33	28		
Close	12	9	0.20	0.91
Positive	9	9		
**Cohesive Front**				
Cohesive	104	50	18.01	**<0.0001**
Discohesive	42	61		
**Primary Treatment Modality**				
Surgery	52	45		
(Chemo)radiation	86	55	9.06	**0.03**
Palliative	4	12		
**Radiotherapy Received?**				
None	9	30		
Primary	85	52	21.42	**<0.0001**
Adjuvant	52	29		

χ^2 statistic is displayed together with *p*-values. Significant *p*-values (<0.05) are shown in bold type.

Table S2. Univariate analysis results for OPSCC patients.

Category	All OPSCC		HPV POSITIVE		HPV NEGATIVE	
	Univariate HR (95% CI)	*p* -value	Univariate HR (95% CI)	*p* -value	Univariate HR (95% CI)	*P*-value
	**Age**					
For each additional year	1.03 (1.02–1.05)	**<0.0001**	1.04 (1.01–1.07)	**0.003**	1.03 (1.01–1.05)	**0.003**

**HPV status**						
HPV^{+ve}	1					
HPV^{-ve}	2.72 (1.88–3.94)	**<0.0001**				
**Gender**						
Female	1		1		1	
Male	1.34 (0.88–2.05)	0.17	1.73 (0.83–3.6)	0.14	1.26 (0.75–2.12)	0.38
**Smoking**						
Non smoker/Ex smoker/<10 pack year	1		1		1	
>10 pack years	1.78 (1.16–2.72)	**0.01**	2.16 (1.11–4.22)	**0.02**	0.71 (0.41–1.25)	0.24
**Alcohol**						
Non-drinker/Ex drinker	1		1		1	
Current drinker	0.85 (0.50–1.47)	0.56	0.94 (0.39–2.27)	0.9	0.79 (0.40–1.59)	0.52
**Tumour Stage**						
Tis/T1/T2	1		1		1	
T3/T4	2.36 (1.63–3.44)	**<0.0001**	2.94 (1.64–5.29)	**<0.0001**	1.74 (1.07–2.84)	**0.03**
**N Stage**						
N0-N2a	1		1		1	
N2b-N3	1.03 (0.70–1.52)	0.89	1.11 (0.56–2.19)	0.77	1.73 (1.05–2.82)	**0.03**
**Treatment**						
Surgery	1		1		1	
(Chemo)radiation	1.38 (0.92–2.08)	0.13	1.52 (0.76–2.98)	0.22	1.54 (0.91–2.60)	0.11
**Cten Score**						
Absent/low	1		1		1	
Moderate/high	2.94 (1.97–4.38)	**<0.0001**	3.30 (1.81–6.01)	**<0.0001**	1.65 (0.95–2.85)	0.073

Full survival data available in n = 259 and presented both as total cohort and subdivided by HPV status. Hazard ratios (HR) are displayed together with the 95% confidence intervals. Significant p-values (<0.05) are shown in bold type. HPV^{-ve} status, current smoking (>10 pack years) and advanced T-stage were all significant on univariate analysis in the total cohort and included in a multivariate model to examine CTEN expression as an independent prognostic variable. CTEN expression demonstrated significance on univariate ($p < 0.0001$) and subsequent multivariate analysis ($p < 0.0001$). Tis = carcinoma in situ.

Table S3. Legend map for Proteome Profiler Human Apoptosis Array Kit (R&D Systems, MN, USA).

Coordinate	Target/Control	Coordinate	Target/Control
A1, A2	Reference Spots	C13, C14	HO-2/HMOX2
A23, A24	Reference Spots	C15, C16	HSP27
B1, B2	Bad	C17, C18	HSP60
B3, B4	Bax	C19, C20	HSP70
B5, B6	Bcl-2	C21, C22	HTRA2/Omi
B7, B8	Bcl-x	C23, C24	Livin
B9, B10	Pro-Caspase-3	D1, D2	PON2
B11, B12	Cleaved Caspase-3	D3, D4	p21/CIP1/CDKN1A
B13, B14	Catalase	D5, D6	p27/Kip1
B15, B16	cIAP-1	D7, D8	Phospho-p53 (S15)
B17, B18	cIAP-2	D9, D10	Phospho-p53 (S46)
B19, B20	Claspin	D11, D12	Phospho-p53 (S392)
B21, B22	Clusterin	D13, D14	Phospho-Rad17 (S635)
B23, B24	Cytochrome c	D15, D16	SMAC/Diablo
C1, C2	TRAIL R1/DR4	D17, D18	Survivin

C3, C4	TRAIL R2/DR5	D19, D20	TNF RI/TNFRSF1A
C5, C6	FADD	D21, D22	XIAP
C7, C8	Fas/TNFRSF6/CD95	D23, D24	PBS (Negative Control)
C9, C10	HIF-1α	E1, E2	Reference Spots
CC12	HO-1/HMOX1/HSP32		

Test comprises a membrane-fixed antibody array for relative protein expression analysis in cell lysates. Result from the present study with membrane array are displayed in Figure 4b.

© 2020 by the authors. Licensee MDPI, Basel, Switzerland. This article is an open access article distributed under the terms and conditions of the Creative Commons Attribution (CC BY) license (http://creativecommons.org/licenses/by/4.0/).
